# Genotype and biotype of invasive *Anopheles stephensi* in Mannar Island of Sri Lanka

**DOI:** 10.1186/s13071-017-2601-y

**Published:** 2018-01-03

**Authors:** Sinnathamby N. Surendran, Kokila Sivabalakrishnan, Kanapathy Gajapathy, Sivasingham Arthiyan, Tibutius T. P. Jayadas, Kalingarajah Karvannan, Selvarajah Raveendran, S. H. P. Parakrama Karunaratne, Ranjan Ramasamy

**Affiliations:** 1 Department of Zoology, University of Jaffna, Jaffna, Sri Lanka; 2 Department of Geography, University of Jaffna, Jaffna, Sri Lanka; 3 Department of Zoology, University of Peradeniya, Peradeniya, Sri Lanka; 4 ID-FISH Technology Inc., 797 San Antonio Road, Palo Alto, CA 94303 USA

**Keywords:** *Anopheles stephensi*, Biotype, *cox*1, *cytb*, Malaria vector, Mannar Island, Spiracular index, Sri Lanka, ITS2

## Abstract

**Background:**

*Anopheles stephensi*, the major vector of urban malaria in India, was recently detected for the first time in Sri Lanka in Mannar Island on the northwestern coast. Since there are different biotypes of *An. stephensi* with different vector capacities in India, a study was undertaken to further characterise the genotype and biotype of *An. stephensi* in Mannar Island.

**Methods:**

Mosquito larvae were collected in Pesalai village in Mannar and maintained in the insectary until adulthood. Adult *An. stephensi* were identified morphologically using published keys. Identified adult *An. stephensi* were molecularly characterized using two mitochondrial (*cox*1 and *cytb*) and one nuclear (ITS2) markers. Their PCR-amplified target fragments were sequenced and checked against available sequences in GenBank for phylogenetic analysis. The average spiracular and thoracic lengths and the spiracular index were determined to identify biotypes based on corresponding indices for Indian *An. stephensi*.

**Results:**

All DNA sequences for the Mannar samples matched reported sequences for *An. stephensi* from the Middle East and India. However, a single nucleotide variation in the *cox*1 sequence suggested an amino acid change from valine to methionine in the *cox*1 protein in Sri Lankan *An. stephensi*. Morphological data was consistent with the presence of the Indian urban vector *An. stephensi* type-form in Sri Lanka.

**Conclusions:**

The present study provides a more detailed molecular characterization of *An. stephensi* and suggests the presence of the type-form of the vector for the first time in Sri Lanka. The single mutation in the *cox*1 gene may be indicative of a founder effect causing the initial diversification of *An. stephensi* in Sri Lanka from the Indian form. The distribution of the potent urban vector *An. stephensi* type-form needs to be established by studies throughout the island as its spread adds to the challenge of maintaining the country’s malaria-free status.

**Electronic supplementary material:**

The online version of this article (10.1186/s13071-017-2601-y) contains supplementary material, which is available to authorized users.

## Background

Malaria was eliminated from Sri Lanka in 2013 but has probably been endemic in the country since the thirteenth century [1]. However, 36–95 cases of imported malaria per year have been detected in returning residents and visitors to the country after 2013 [1]. Because high densities of many anopheline vectors are present on the island, continuing vigilance to prevent the re-initiation of indigenous malaria transmission from externally acquired infections is therefore necessary. *Anopheles culicifacies* species E is the established primary vector of malaria in Sri Lanka with *An. subpictus*, *An. sundaicus* and *An. annularis* as secondary vectors together with several other minor vectors [2–6]. *Anopheles stephensi*, never previously detected in Sri Lanka [7, 8], was recently identified on the island of Mannar off the northwestern coast of Sri Lanka [9].

*Anopheles stephensi* is a major malaria vector in the Indian subcontinent and is widely distributed in Asia, being present in India, Pakistan, Afghanistan, Iran, Iraq, Bahrain, Oman and Saudi Arabia in the West to Bangladesh, South China and Myanmar in the East [10, 11]. It is a major vector of urban malaria on the Indian subcontinent and the Middle East [12–14].

Although *An. stephensi* is not considered to be a species complex, three different biotypes have been identified in India namely type, intermediate and *mysorensis*, based on morphological differences in the number of egg ridges [15, 16], spiracular index [17] cytogenetic characteristics [18, 19], cuticular hydrocarbon profiles [20], as well as through differences in ecological, behavioral and mating characteristics [11]. The type*-*form is considered to be a vector of urban malaria, while the *mysorensis*-form is considered to be a poor vector or non-vector due to its zoophagic nature [16]. The vector status of the intermediate form is not well-established.

After the detection of *An. stephensi* on Mannar Island [9], strict adult and preimaginal vector control measures have been implemented by the Antimalaria Campaign (AMC) to eliminate it and curtail its spread to the mainland. The initial identification of *An. stephenisi* in Mannar was based on larval and adult morphology and cytochrome oxidase or *cox*1 sequence [9]. The present study was undertaken to further molecular characterize *An. stephensi* on Mannar Island using an additional mitochondrial gene cytochrome b (*cytb)*, and the nuclear internally transcribed spacer 2 of ribosomal RNA (ITS2), and also to morphologically characterize the biotypes.

## Methods

### Study site

**Fig. 1:**
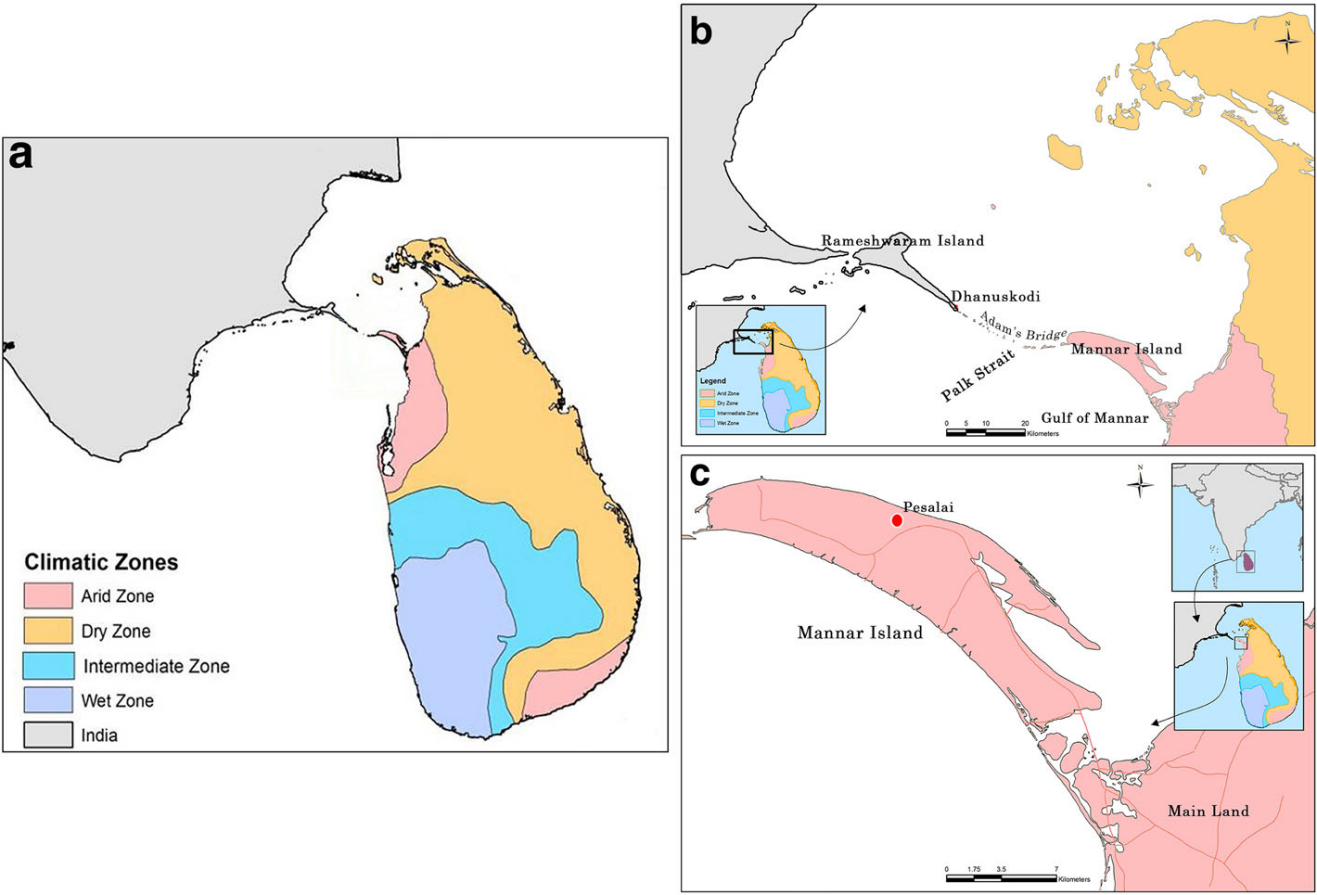
The map of **a** location of Sri Lanka in the Indian Ocean and its four rainfall zones; **b** location of Mannar Island relative to Tamil Nadu; and **c** location of the sample collection site Pesalai in Mannar Island

Mannar Island lies in the Indian ocean off the Northwestern coast of Sri Lanka in close proximity (*c.*30 km) to Rameshwaram Island of the state of Tamil Nadu in India (Fig. 1). Mannar and Rameshwaram islands are separated by a shallow region of the Gulf of Mannar called the Palk Strait (Fig. 1) made up of chains of sandbanks and islets. Mannar Island is located within one of the two arid zones of Sri Lanka that receives average rainfall of < 800 mm rain annually (Fig. 1). This contrasts with the average annual rainfall in the wet zone of 2500 mm and the dry zone of <1900 mm and an intermediate zone with mixed characteristics [21]. Larval collections were performed in Pesalai, one of many rural villages of Mannar district where the presence of *An. stephensi* was first discovered [9]. Fishing is the main occupation in Pesalai.

### Sample collection and species identification

Anopheline larvae were collected from domestic wells and cement tanks identified as potential habitats for *An. stephensi* during the dry season months of June-August, 2017 using standard dippers (350 ml). Collected larvae were reared as described previously in the contained insectary conditions of the Department of Zoology, University of Jaffna to reach adulthood [22]. Larvae were maintained under laboratory conditions (28 ± 2 °C, 12 h photoperiodicity and RH ~70%) in the same water from the habitats in which they were collected, in 1.5 l plastic trays with powered fish meal given twice a day as additional food. The emergent adults were identified morphologically as *An. stephensi* using published keys [7, 23] and used for subsequent molecular characterization and biotype identification.

### PCR amplification of target molecular markers

DNA from 25 individuals morphologically identified as *An. stephensi* was extracted using the DNeasy Blood & Tissue kit (Qiagen, California, USA). The ITS2 region of rDNA was amplified using the 5.8S forward and 28S reverse primers [24]. Regions of the *cox*1 and *cytb* genes of mitochondria were amplified by using primers C1-J-1718 and C1-N-2191 [25] and cytbF and cytbR [26], respectively. For each amplification, PCR reactions were performed in a 25 μl volume as described previously [27]. The PCR products were purified using QIAquick® PCR Purification Kit (Qiagen). Purified PCR products of ITS2, *cox*1, and *cytb* were sequenced in both directions at the sequencing facility of the University of Peradeniya, Peradeniya, Sri Lanka.

### Sequence analysis and phylogenetic analysis

The sequences were edited in Finch TV (Geospiza Inc., Seattle, USA) and aligned with Clustal W in MEGA 5.0 software [28]. The aligned sequences for coding genes were translated into amino acid sequences using the invertebrate mitochondrial codon usage pattern. The amino acid sequences were used to find out the best matching protein in BLASTP of the NCBI. Neighbour-joining trees were created with the best substitution model selected from MEGA 5.0 [28]. For *cox*1 sequences based phylogenetic analysis the best model selected was Tamura Nei 92 with the rate parameters set at gamma distribution with invariant sites and the Jukes-Cantor model for ITS2 with uniform rate distribution. One thousand nonparametric bootstrap replicates were performed and a consensus tree was constructed. The DNA sequences were submitted in the GenBank database under the accession numbers MF975722–MF975748. The uncorrected Kimura 2-Parameter (K2P) genetic distance [28] was calculated using MEGA 5.0. DNA sequence polymorphism such as number of haplotypes was estimated using DnaSP 5.10 [29].

### Biotype identification based on spiracular index

Morphologically identified adult *An. stephensi* from Pesalai were used for determining their biotype based on the spiracular index as described by Nagpal et al. [17] for Indian *An. stephensi* type-form and *An. stephensi* var. *mysorensis*. Essentially the adults were kept in 10% KOH overnight and the spiracles and thorax were dissected under a binocular dissecting microscope (Kyowa, Kanagawa, Japan) and mounted in Canada balsam. The length of spiracles and thorax were measured using a light microscope (Olympus - CX21, Tokyo, Japan) equipped with ocular and stage micrometers. The spiracular index was calculated (mean spiracular length divided by thoracic length × 100) as described previously [30]. The calculated spiracular index was compared with the reported indices for Indian type- and *mysorensi*s-forms [17] and subjected to a two-tailed Student’s t-test to identify significant differences.

## Results

### Sample collection and species identification

During the study period, 24 potential habitats (cemented water storage tanks, open wells and domestic wells) were inspected and 11 were found to have mosquito larvae. Collections were limited to larvae from Pesalai due to strict control measures implemented by the regional AMC with the application of Temephos to many larval habitats. Out of the 11 sites with mosquito larvae, 7 sites were found to have a total of 158 anopheline larvae. Of the emergent adults 73 were identified as *An. stephensi* and the rest as *Anopheles varuna*. All the 7 preimaginal habitats that contained *An. stephensi* were domestic wells where the water was used for purposes other than drinking.

### DNA sequence analysis

A fragment size of 590 bp sequence was obtained for *cox*1 and used for analysis with other sequences retrieved from GenBank. A total of nine *cox*1 sequences were retrieved from GenBank and aligned with ten sequences obtained from the present study. A fragment size of 347 bp sequence of *cox*1 gene which had maximum hits in the NCBI nucleotide BLASTn search was used for the analysis. The *cox*1 sequences were free of nuclear gene copies and checked with the reference genome of *An. stephensi* for consistency. No deletions, insertions or stop codons were observed. This indicates the absence of pseudogenes. All analyzed sequences were overlapping and covered the same region. During the alignment all the sequences available from GenBank, no variations were observed except that four samples (three from the present study GenBank accession numbers MF975729–31 and one from a previous submission MF124611) from Sri Lanka had a single nucleotide variation. The variation was a transition where a G is replaced by an A (Additional file 1: Figure S1). The variation causes a change in the amino acid sequence where valine is replaced by methionine (Additional file 2: Figure S2). The calculated K2P distance among the group was within 0.3 and 0.6%.

Three *cox*1 haplotypes were identified with the dominant form having fourteen samples (including seven sequences from the current study; GenBank: MF975722–28), another haplotype with four samples (three of the present study; GenBank: MF 975729–31), one from a previously reported sequence from Sri Lanka (GenBank: MF124611) and one with a single sequence of a sample collected from Pondicherry, India (GenBank: DQ310148).

A PCR-amplified 230 bp ITS2 region was analyzed with 15 sequences obtained from GenBank in addition to the 11 sequences from the present study. The ITS2 sequences confirmed the identity of the species with no variation observed among the identified samples from our study. The aligned sequence shows some degree of variation among other sequence reported from Iran, India, Saudi Arabia and Iraq. A total of five haplotypes were identified based on ITS2 sequence data. All the samples from the current study was categorized into one haplotype (MF975738–48), and identical to the Indian sample collected from Tamil Nadu state in India (GenBank: EU359681) [31] whereas the other four haplotypes had one sequence each from India (KT921409), Iran (DQ662409 and AY702485) and one without any location identifier (AY702485). The K2P distance calculated was 0.5 to 27.2% among the group with the sample with the accession number of HQ703001 from India showing greater variation from the others.

A fragment size of 232 bp was generated for *cytb* sequences and were deposited in GenBank (MF975732–37). The identity of the species was confirmed by the *cytb* sequence analysis with available sequences in GenBank (AF311254 and KT382827). There were no variations found in the *cytb* sequences within the Sri Lanka samples and the two sequences (AF311254 and KT382827) deposited in GenBank. The tree analysis for the *cytb* was not done as there were only two other sequences available in GenBank.

### Maximum likelihood tree

**Fig. 2:**
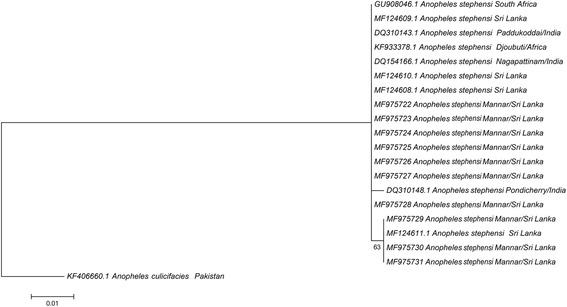
Phylogenetic tree based on *cox*1 sequence dataset (347 nt positions) constructed using the maximum likelihood method using Tamura-Nei 92 model with the rate parameters set at gamma distribution with invariant sites; bootstrap values > 63% are shown. The sequences used for analysis include samples from Pesalai (GenBank: MF975722–MF975731) and other GenBank entries. *Anopheles culicifacies* (GenBank: KF406660) was used as the outgroup

**Fig. 3:**
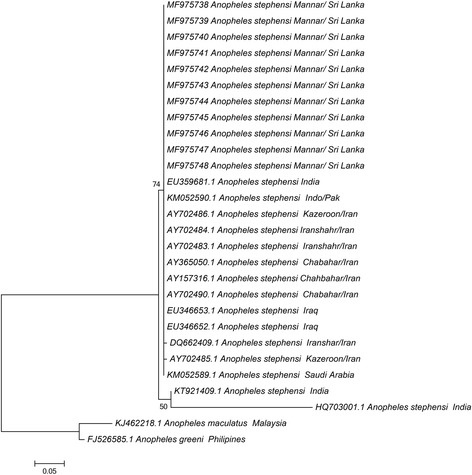
Phylogenetic tree based on ITS2 sequence dataset (230 nt positions) constructed using the maximum likelihood method using the Jukes-Cantor model; bootstrap values > 50% are shown. The sequences used for analysis include the samples from Pesalai (GenBank: MF975738–MF975748) and other GenBank entries. *Anopheles maculatus* (GenBank: KJ462218) and *An. greeni* (GenBank: FJ526585) were used as the outgroup

The phylogeny trees created using *cox*1 and ITS2 sequence data are presented in Figs. 2 and 3, respectively. Only one base difference was present within the Sri Lankan group for *cox*1. The other *cox*1 sequences also did not vary enough to create separate clades with strong bootstrap values. Instead they are all grouped as a single clade. The phylogenetic analysis based on ITS2 sequence data resulted in 3 separate clades. All except two different sequences were found to be grouped as a single clade. Two from India representing Rajasthan (GenBank: KT921409) and an unknown locality (GenBank: HQ703001) were separated from each other and rest of the samples with strong bootstrap values.

### Identification of biotype

Thirty-two adults emerging from larvae collected in Pesalai were used for determining biotype. The average spiracular length varied between 0.10–0.12 mm and the thoracic length varied between 1.04–1.25 mm. The spiracular index varied between 8.19–10.10. The mean values of spiracular length, thoracic length and spiracular index are shown in Table 1. The analysis based on spiracular index in comparison with the corresponding reported index for Indian samples [17] were consistent with all 32 Pesalai specimens analyzed belonging to type biological form. The Student’s t-test analysis revealed no significant (*t*_(62)_ = 0.69, *P* = 0.491) difference between the Indian and Pesalai type-forms and a significant (*t*_(62)_ = 15.78, *P* = 0.005) difference between the Indian *mysorensis-* and Pesalai type*-*form.

**Table 1:** Measurements of spiracular length, thoracic length and calculated spiracular index of adult *An. stephensi*

Country/location	Mean spiracular length (mm)	Mean thoracic length (mm)	Mean spiracular index	Biotype
Pesalai/Sri Lanka	0.11 ± 0.01	1.14 ± 0.06	9.32 ± 0.53^a^	type
Jodhpur/India [22]	0.12 ± 0.01	1.30 ± 0.08	9.23 ± 0.51^a^	type
Jodhpur/India [22]	0.10 ± 0.01	1.33 ± 0.05	7.52 ± 0.34	*mysorensis*

^a^Difference not statistically significant

## Discussion

The recently identified *An. stephensi* on Mannar Island, Sri Lanka has been now characterized with three different genetic markers. The DNA sequence analysis of the three marker genes showed that *An. stephensi* collected from Pesalai are genetically identical to each other in the three markers, and that they are related to *An. stephensi* populations in the Middle East and Indian subcontinent. However, a single nucleotide variation that resulted in an amino acid variation (Val to Met) characteristic to Sri Lankan *An. stephensi cox*1 subunit is reported here for the first time. This may be indicative of a founder effect resulting from a limited invasion into Mannar. The specimens are unlikely to be derived from eggs from the same blood-fed female as the larvae were collected from seven different wells in Pesalai.

To our knowledge, this is also the first indication that the efficient urban vector biological form of *An. stephensi*, the type-form, is present in Mannar Island in Sri Lanka. The limitations of the present study were that the samples were collected from a single location and blood-fed adult *An. stephensi* were not available due to the vector control measures implemented by the AMC throughout Mannar Island. Additional studies on the biotype and genotype may become possible if self-mating colonies of *An. stephensi* originating in Sri Lanka can be established. Larval collections to determine the presence of *An. stephensi* in other locations in Sri Lanka also need to be pursued in this context.

Molecular characterization of all three biological forms of Iran using *cox*1 and *cox*2 markers revealed high homology in the sequences suggesting extensive gene flow [14]. Similarly in India, the type- and *mysorensis-*form exhibited sequence similarity in the ribosomal DNA ITS2 and domain-3 [31]. Presence of the three biological forms with sequence identity confirms their taxonomic status and the reported differences in vectorial capacities between biotypes can be attributed to epidemiological factors rather than morphological differences [32]. However, genetic studies based on microsatellite markers revealed the presence of genetic differences and a non-significant low level of gene flow between the three biological forms [33]. Therefore extensive DNA-based studies along with bio-ecological characterizations are needed to establish whether the three forms are genetically distinct populations with different vectorial capacities.

The type-form is reported to be an efficient urban vector responsible for urban malaria in the Indian subcontinent [11]. The type*-*form specimens were found to develop mainly in overhead tanks, cemented tanks, domestic wells, cisterns, fountains and water collections at construction sites [34–36]. However, in contrary to the reports that the type-form is not prevalent in rural environments [11], the type-form found in Pesalai, Sri Lanka, has established its populations in a rural environment to lay eggs and undergo preimaginal development in domestic wells. This adaptation poses a challenge in Sri Lanka as this form of *An. stephensi* has the potential to transmit malaria in both rural and urban environments, especially in the mainland of North Sri Lanka where almost every household has a domestic well.

The spread of *An. stephensi* southward in India has been attributed to be the cause for recent outbreaks of malaria in the normally malaria-free Kerala state [37]. Continuing southward expansion of *An. stephensi* has resulted in the invasion of Lakshadweep islands in the Indian Ocean [37]. The spread was associated with the availability of larval habitats in the form of water storage cement tanks. It was postulated that, after having reached the southern-most areas of India, *An. stephensi* may spread across the narrow Palk Strait to Sri Lanka [37]. The present observations and an earlier report [9] confirm that this has indeed occurred. The spread of *An. stephensi* southward in India and into Sri Lanka, utilizing cemented wells as habitats, may provide an example of the postulated anthropogenically-induced adaptation to invade in mosquito vectors [38]. There has always been a regular movement of people between Mannar and Rameshwaram islands. It is likely that the vector could have arrived from India in water carried in boats to establish itself in the Mannar Island of Sri Lanka.

The identities in *cox*1, *cytb* and ITS2 sequences within the Mannar isolates and the single amino acid change in *cox*1 compared to Indian *An. stephensi* are consistent with a founder effect i.e. the Mannar mosquitoes being derived from a very small number of *An. stephensi* that arrived from India. The amino acid change in *cox*1, indicative of the diversification of Mannar *An. stephensi* from the Indian form, may result in the spread of the mutation in Sri Lanka if *An. stephensi* is not eradicated by the new vector control measures now in place. The movement of people between India and the Jaffna peninsula in North Sri Lanka has also been common, becoming particularly prominent during the civil war of 1983–2009. It remains possible that *An. stephensi* is also present elsewhere in coastal Sri Lanka, particularly the Jaffna peninsula, and has not been detected in the limited entomological surveys performed to date.

## Conclusions

The presence of *An. stephensi* type-form in a rural environmental is of adaptive significance and an added challenge to prevent the re-introduction of malaria into Sri Lanka. Further molecular and bio-ecological studies are warranted to establish the origin, diversity and spread of *An. stephensi* in Sri Lanka.

## Additional files

Additional file 1: Figure S1. Chromatograms of two sequences of *cox*1 show G-A transitions in the Sri Lankan samples. (DOCX 242 kb)

Additional file 2: Figure S2. Amino acid sequence variation in *cox*1 showing the replacement of valine by methionine in the Sri Lankan samples. (RTF 41 kb)

## Abbreviations

AMC: Antimalaria campaign
*cox*1: Cytochrome *c* oxidase subunit 1 gene
cytb: Cytochrome *b* gene
ITS2: Internal transcribed spacer 2

## References

[1] Wijesundere DA, Ramasamy R (2017). Analysis of historical trends and recent elimination of malaria from Sri Lanka and its applicability for malaria control in other countries. Front Public Health.

[2] Amerasinghe PH, Amerasinghe FP, Wirtz RA, Indrajith NG, Somapala W, Pereira LR, Rathnayake AM (1992). Malaria transmission by *Anopheles subpictus* (Diptera: Culicidae) in a new irrigation project in Sri Lanka. J Med Entomol.

[3] Amerasinghe PH, Amerasinghe FP, Konradsen F, Fonseka KT, Wirtz RA (1999). Malaria vectors in a traditional dry zone village in Sri Lanka. Am J Trop Med Hyg..

[4] Ramasamy R, De Alwis R, Wijesundere A, Ramasamy MS (1992). Malaria transmission at a new irrigation project in Sri Lanka: the emergence of *Anopheles annularis* as a major vector. Am J Trop Med Hyg..

[5] Surendran SN, Ramasamy R (2010). The *Anopheles culicifacies* and *An. subpictus* species complexes in Sri Lanka and their implications for malaria control in the country. Trop Med Health.

[6] Surendran SN, Singh OP, Jude PJ, Ramasamy R (2010). Genetic evidence for malaria vectors of the *Anopheles sundaicus* Complex in Sri Lanka with morphological characteristics attributed to *Anopheles subpictus* species B. Malar J.

[7] Amerasinghe FP (1990). A guide to the identification of the anopheline mosquitoes (Diptera: Culicidae) of Sri Lanka -I adult females. Cey J Sci (Biological Science).

[8] Weeraratne TC, Surendran SN, Reimer LJ, Charles S, Wondji CS (2017). Molecular characterization of Anopheline (Diptera: Culicidae) mosquitoes from eight geographical locations of Sri Lanka. Malar J.

[9] Dharmasiri AGG, Perera AY, Harishchandra J, Herath H, Aravindan K, Jayasooriya HRT (2017). First record of *Anopheles stephensi* in Sri Lanka: a potential challenge for prevention of malaria reintroduction. Malar J.

[10] Gakhar SK, Sharma R, Sharma A (2013). Population genetic structure of malaria vector *Anopheles stephensi* Liston (Diptera: Culicidae). Indian J Exp Biol.

[11] World Health Organization (2007). Anopheline species complexes in south and South-East Asia. SEARO technical publication no. 57.

[12] Sharma RS (1995). Urban malaria and its vectors *Anopheles stephensi* and *Anopheles culicifacies* (Diptera: Culicidae) in Gurgaon, India. Southeast Asian J Trop Med Public Health.

[13] Nalin DR, Mahood F, Rathor H, Muttab A, Sakai R, Chowdhary MA (1985). A point survey of periurban and urban malaria in Karachi. J Trop Med Hyg.

[14] Oshaghi MA, Yaaghoobi F, Abaie MR (2006). Pattern of mitochondrial DNA variation between and within *Anopheles stephensi* (Diptera: Culicidae) biological forms suggests extensive gene flow. Acta Trop.

[15] Sweet WC, Rao B (1937). Races of *Anopheles stephensi* Liston. 1901. Ind Med Gaz.

[16] Subbarao SK, Vasantha K, Adak T, Sharma VP, Curtis CF (1987). Egg-float ridge number in *Anopheles stephensi*: ecological variation and genetic analysis. Med Vet Entomol.

[17] Nagpal BN, Srivastava A, Kalra NL, Subbarao SK (2003). Spiracular indices in *Anopheles stephensi*: a taxonomic tool to identify ecological variants. J Med Entomol.

[18] Coluzzi M, Di Deco M, Cancrini G (1973). Chromosomal inversions in *Anopheles stephensi*. Parassitologia.

[19] Suguna SG (1992). Y-chromosome dimorphisms in the malaria vector *Anopheles stephensi* from south India. Med Vet Entomol.

[20] Anyanwu GI, Davies DH, Molyneux DH, Phillips A, Milligan PJ (1993). Cuticular hydrocarbon discrimination/variation among strains of the mosquito, *Anopheles* (*Cellia*) *stephensi* Liston. Ann Trop Med Parasitol.

[21] Ramasamy R, Surendran SN (2012). Global climate change and its potential impact on disease transmission by salinity-tolerant mosquito vectors in coastal zones. Front Physiol.

[22] Jude PJ, Dharshini S, Vinobaba M, Surendran SN, Ramasamy R (2010). *Anopheles culicifacies* breeding in brackish waters in Sri Lanka and implications for malaria control. Malar J.

[23] Nagpal BN, Srivastava A, Saxena R, Ansari MA, Dash AP, Das SC (2005). Pictorial identification key for Indian anophelines.

[24] Collins FH, Paskewitz SM (1996). A review of the use of ribosomal DNA (rDNA) to differentiate among cryptic *Anopheles* species. Insect Mol Biol.

[25] Simon C, Frati F, Beckenbach A, Crepsi B, Liu H, Flook P (1994). Evolution, weighting and phylogenetic utility of mitochondrial gene sequences and a compilation of conserved polymerase chain reaction primers. Ann Entomol Soc Am.

[26] Lyman DF, Monteiro FA, Escalante AA, Cordon-Rosales C, Wesson DM, Dujardin JP, Beard CB (1999). Mitochondrial DNA sequence variation among triatomine vectors of Chagas' disease. Am J Trop Med Hyg.

[27] Surendran NS, Sarma DK, Jude PJ, Kemppainen P, Kanthakumaran N, Gajapathy K (2013). Molecular characterization and identification of members of the *Anopheles subpictus* Complex in Sri Lanka. Malar J.

[28] Tamura K, Peterson D, Peterson N, Stecher G, Nei M, Kumar S (2011). MEGA5: molecular evolutionary genetics analysis using maximum likelihood, evolutionary distance, and maximum parsimony methods. Mol Biol Evol.

[29] Librado P, Rozas J (2009). DnaSP v5: a software for comprehensive analysis of DNA polymorphism data. Bioinformatics.

[30] Vinogradaskaya ON (1969). Geographic distribution of mosquitoes - vectors of infections (on the basis of their xerophilic and hygrophilly).

[31] Alam MT, Bora H, Das MK, Sharma YD (2008). The type and mysorensis forms of the *Anopheles stephensi* (Diptera: Culicidae) in India exhibit identical ribosomal DNA ITS2 and domain-3 sequences. Parasitol Res.

[32] Chavshin AR, Oshaghi MA, Vatandoost H, Hanafi-Bojd AA, Raeisi A, Nikpoor F (2014). Molecular characterization, biological forms and sporozoite rate of *Anopheles stephensi* in southern Iran. Asian Pac J Trop Biomed.

[33] Vipin S, Dube M, Gakhar SK (2010). Genetic differentiation between three ecological variants (‘type’, ‘mysorensis’ and ‘intermediate’) of malaria vector *Anopheles stephensi* (Diptera: Culicidae). Insect Sci.

[34] Rao TR (1984). The anophelines of India.

[35] Mariappan T, Thenmozhi V, Udayakumar P, Bhavaniumadevi V, Tyagi BK. An observation on breeding behaviour of three different vector species (*Aedes aegypti* Linnaeus, 1762, *Anopheles stephensi* Liston, 1901 and *Culex quinquefasciatus* Say, 1823) in wells in the coastal region of Ramanathapuram district, Tamil Nadu, India. Int J Mosq Res. 2015;2(2):42–4.

[36] Thomas S, Ravishankaran S, Justin JA, Asokan A, Mathai MT, Valecha N (2016). Overhead tank is the potential breeding habitat of *Anopheles stephensi* in an urban transmission setting of Chennai, India. Malar J.

[37] Sharma SK, Hamzakoya KK (2001). Geographical spread of *Anopheles stephensi*, vector of urban malaria, and *Aedes aegypti*, vector of dengue/DHF, in the Arabian Sea islands of Lakshadweep, India. Dengue Bulletin.

[38] Ramasamy R, Surendran SN (2016). Mosquito vectors developing in atypical anthropogenic habitats - global overview of recent observations, mechanisms and impact on disease transmission. J Vector Borne Dis.

