# Supplementary figures and images for: Genotype and biotype of invasive Anopheles stephensi in Mannar Island of Sri Lanka
Source: Parasit Vectors. 2018 Jan 3;11:3. doi: 10.1186/s13071-017-2601-y (PMC5753456; doi:10.1186/s13071-017-2601-y)

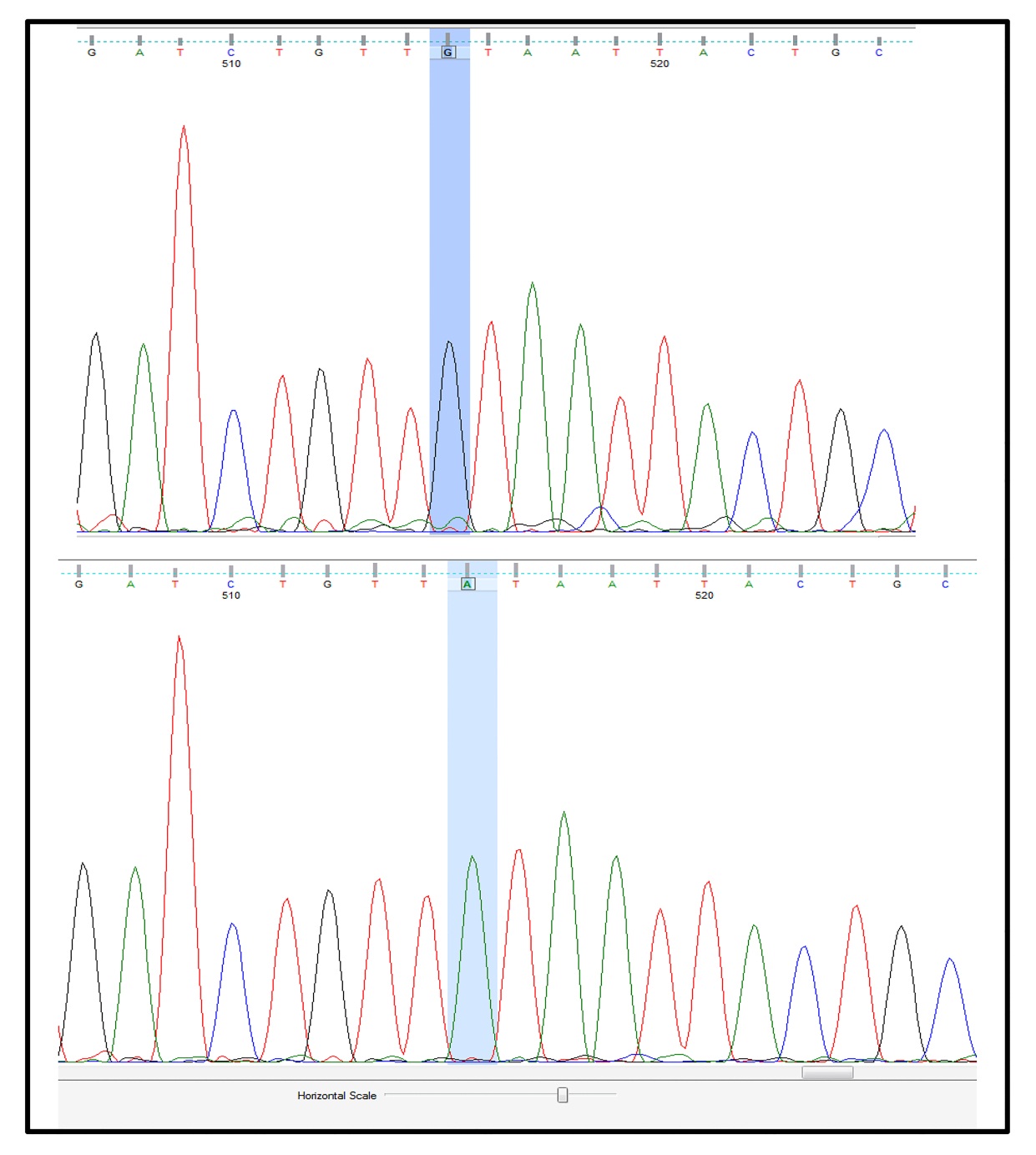


**Figure S1.** Chromatograms of two sequences of *cox*1 show G–A transitions in the Sri Lankan samples.

Supplement: Supplementary file 1 — Chromatograms of two sequences of cox1 show G-A transitions in the Sri Lankan samples. (DOCX 242 kb) [file 13071_2017_2601_MOESM1_ESM.docx]
