# Supplementary material for: Genotype and biotype of invasive Anopheles stephensi in Mannar Island of Sri Lanka
Source: Parasit Vectors. 2018 Jan 3;11:3. doi: 10.1186/s13071-017-2601-y (PMC5753456; doi:10.1186/s13071-017-2601-y)
Supplement: Supplementary file 2 — Amino acid sequence variation in cox1 showing the replacement of valine by methionine in the Sri Lankan samples. (RTF 41 kb) [file 13071_2017_2601_MOESM2_ESM.rtf]

Figure S2. Amino acid sequence variation in cox1 showing the replacement of valine is by methionine in the Sri Lankan samples

JFN_ZOO_AN.STEPHENSI_MANNAR_F_00  LYFIFGAWAGMVGTSLSILI [ 20]
JFN_ZOO_AN.STEPHENSI_MANNAR_F_00  .................... [ 20]
JFN_ZOO_AN.STEPHENSI_MANNAR_F_00  .................... [ 20]
JFN_ZOO_AN.STEPHENSI_MANNAR_M_00  .................... [ 20]
JFN_ZOO_AN.STEPHENSI_MANNAR_M_00  .................... [ 20]
JFN_ZOO_AN.STEPHENSI_MANNAR_F_00  .................... [ 20]
JFN_ZOO_AN.STEPHENSI_MANNAR_F_00  .................... [ 20]
JFN_ZOO_AN.STEPHENSI_MANNAR_M_00  .................... [ 20]
JFN_ZOO_AN.STEPHENSI_MANNAR_F_00  .................... [ 20]
JFN_ZOO_AN.STEPHENSI_MANNAR_M_00  .................... [ 20]

JFN_ZOO_AN.STEPHENSI_MANNAR_F_00  RAELGHPGAFIGDDQIYNVI [ 40]
JFN_ZOO_AN.STEPHENSI_MANNAR_F_00  .................... [ 40]
JFN_ZOO_AN.STEPHENSI_MANNAR_F_00  .................... [ 40]
JFN_ZOO_AN.STEPHENSI_MANNAR_M_00  .................... [ 40]
JFN_ZOO_AN.STEPHENSI_MANNAR_M_00  .................... [ 40]
JFN_ZOO_AN.STEPHENSI_MANNAR_F_00  .................... [ 40]
JFN_ZOO_AN.STEPHENSI_MANNAR_F_00  .................... [ 40]
JFN_ZOO_AN.STEPHENSI_MANNAR_M_00  .................... [ 40]
JFN_ZOO_AN.STEPHENSI_MANNAR_F_00  .................... [ 40]
JFN_ZOO_AN.STEPHENSI_MANNAR_M_00  .................... [ 40]

JFN_ZOO_AN.STEPHENSI_MANNAR_F_00  VTAHAFIMIFFMVMPIMIGG [ 60]
JFN_ZOO_AN.STEPHENSI_MANNAR_F_00  .................... [ 60]
JFN_ZOO_AN.STEPHENSI_MANNAR_F_00  .................... [ 60]
JFN_ZOO_AN.STEPHENSI_MANNAR_M_00  .................... [ 60]
JFN_ZOO_AN.STEPHENSI_MANNAR_M_00  .................... [ 60]
JFN_ZOO_AN.STEPHENSI_MANNAR_F_00  .................... [ 60]
JFN_ZOO_AN.STEPHENSI_MANNAR_F_00  .................... [ 60]
JFN_ZOO_AN.STEPHENSI_MANNAR_M_00  .................... [ 60]
JFN_ZOO_AN.STEPHENSI_MANNAR_F_00  .................... [ 60]
JFN_ZOO_AN.STEPHENSI_MANNAR_M_00  .................... [ 60]

JFN_ZOO_AN.STEPHENSI_MANNAR_F_00  FGNWLVPLMLGAPDMAFPRM [ 80]
JFN_ZOO_AN.STEPHENSI_MANNAR_F_00  .................... [ 80]
JFN_ZOO_AN.STEPHENSI_MANNAR_F_00  .................... [ 80]
JFN_ZOO_AN.STEPHENSI_MANNAR_M_00  .................... [ 80]
JFN_ZOO_AN.STEPHENSI_MANNAR_M_00  .................... [ 80]
JFN_ZOO_AN.STEPHENSI_MANNAR_F_00  .................... [ 80]
JFN_ZOO_AN.STEPHENSI_MANNAR_F_00  .................... [ 80]
JFN_ZOO_AN.STEPHENSI_MANNAR_M_00  .................... [ 80]
JFN_ZOO_AN.STEPHENSI_MANNAR_F_00  .................... [ 80]
JFN_ZOO_AN.STEPHENSI_MANNAR_M_00  .................... [ 80]

JFN_ZOO_AN.STEPHENSI_MANNAR_F_00  NNMSFWMLPPSLTLLISSSM [100]
JFN_ZOO_AN.STEPHENSI_MANNAR_F_00  .................... [100]
JFN_ZOO_AN.STEPHENSI_MANNAR_F_00  .................... [100]
JFN_ZOO_AN.STEPHENSI_MANNAR_M_00  .................... [100]
JFN_ZOO_AN.STEPHENSI_MANNAR_M_00  .................... [100]
JFN_ZOO_AN.STEPHENSI_MANNAR_F_00  .................... [100]
JFN_ZOO_AN.STEPHENSI_MANNAR_F_00  .................... [100]
JFN_ZOO_AN.STEPHENSI_MANNAR_M_00  .................... [100]
JFN_ZOO_AN.STEPHENSI_MANNAR_F_00  .................... [100]
JFN_ZOO_AN.STEPHENSI_MANNAR_M_00  .................... [100]
JFN_ZOO_AN.STEPHENSI_MANNAR_F_00  VENGAGTGWTVYPPLSSGIA [120]
JFN_ZOO_AN.STEPHENSI_MANNAR_F_00  .................... [120]
JFN_ZOO_AN.STEPHENSI_MANNAR_F_00  .................... [120]
JFN_ZOO_AN.STEPHENSI_MANNAR_M_00  .................... [120]
JFN_ZOO_AN.STEPHENSI_MANNAR_M_00  .................... [120]
JFN_ZOO_AN.STEPHENSI_MANNAR_F_00  .................... [120]
JFN_ZOO_AN.STEPHENSI_MANNAR_F_00  .................... [120]
JFN_ZOO_AN.STEPHENSI_MANNAR_M_00  .................... [120]
JFN_ZOO_AN.STEPHENSI_MANNAR_F_00  .................... [120]
JFN_ZOO_AN.STEPHENSI_MANNAR_M_00  .................... [120]

JFN_ZOO_AN.STEPHENSI_MANNAR_F_00  HAGASVDLAIFSLHLAGISS [140]
JFN_ZOO_AN.STEPHENSI_MANNAR_F_00  .................... [140]
JFN_ZOO_AN.STEPHENSI_MANNAR_F_00  .................... [140]
JFN_ZOO_AN.STEPHENSI_MANNAR_M_00  .................... [140]
JFN_ZOO_AN.STEPHENSI_MANNAR_M_00  .................... [140]
JFN_ZOO_AN.STEPHENSI_MANNAR_F_00  .................... [140]
JFN_ZOO_AN.STEPHENSI_MANNAR_F_00  .................... [140]
JFN_ZOO_AN.STEPHENSI_MANNAR_M_00  .................... [140]
JFN_ZOO_AN.STEPHENSI_MANNAR_F_00  .................... [140]
JFN_ZOO_AN.STEPHENSI_MANNAR_M_00  .................... [140]

JFN_ZOO_AN.STEPHENSI_MANNAR_F_00  ILGAVNFITTVINMRSPGIT [160]
JFN_ZOO_AN.STEPHENSI_MANNAR_F_00  .................... [160]
JFN_ZOO_AN.STEPHENSI_MANNAR_F_00  .................... [160]
JFN_ZOO_AN.STEPHENSI_MANNAR_M_00  .................... [160]
JFN_ZOO_AN.STEPHENSI_MANNAR_M_00  .................... [160]
JFN_ZOO_AN.STEPHENSI_MANNAR_F_00  .................... [160]
JFN_ZOO_AN.STEPHENSI_MANNAR_F_00  .................... [160]
JFN_ZOO_AN.STEPHENSI_MANNAR_M_00  .................... [160]
JFN_ZOO_AN.STEPHENSI_MANNAR_F_00  .................... [160]
JFN_ZOO_AN.STEPHENSI_MANNAR_M_00  .................... [160]

JFN_ZOO_AN.STEPHENSI_MANNAR_F_00  LDRMPLFVWSVVITAILLLL [180]
JFN_ZOO_AN.STEPHENSI_MANNAR_F_00  .................... [180]
JFN_ZOO_AN.STEPHENSI_MANNAR_F_00  .................... [180]
JFN_ZOO_AN.STEPHENSI_MANNAR_M_00  .................... [180]
JFN_ZOO_AN.STEPHENSI_MANNAR_M_00  .................... [180]
JFN_ZOO_AN.STEPHENSI_MANNAR_F_00  .................... [180]
JFN_ZOO_AN.STEPHENSI_MANNAR_F_00  .................... [180]
JFN_ZOO_AN.STEPHENSI_MANNAR_M_00  ...........M........ [180]
JFN_ZOO_AN.STEPHENSI_MANNAR_F_00  ...........M........ [180]
JFN_ZOO_AN.STEPHENSI_MANNAR_M_00  ...........M........ [180]


JFN_ZOO_AN.STEPHENSI_MANNAR_F_00  SLPVLAGAITMLLTDR [196]
JFN_ZOO_AN.STEPHENSI_MANNAR_F_00  ................ [196]
JFN_ZOO_AN.STEPHENSI_MANNAR_F_00  ................ [196]
JFN_ZOO_AN.STEPHENSI_MANNAR_M_00  ................ [196]
JFN_ZOO_AN.STEPHENSI_MANNAR_M_00  ................ [196]
JFN_ZOO_AN.STEPHENSI_MANNAR_F_00  ................ [196]
JFN_ZOO_AN.STEPHENSI_MANNAR_F_00  ................ [196]
JFN_ZOO_AN.STEPHENSI_MANNAR_M_00  ................ [196]
JFN_ZOO_AN.STEPHENSI_MANNAR_F_00  ................ [196]
JFN_ZOO_AN.STEPHENSI_MANNAR_M_00  ................ [196]
